# Evolutionary-Related High- and Low-Virulent Classical Swine Fever Virus Isolates Reveal Viral Determinants of Virulence

**DOI:** 10.3390/v16010147

**Published:** 2024-01-19

**Authors:** Yoandry Hinojosa, Matthias Liniger, Obdulio García-Nicolás, Markus Gerber, Anojen Rajaratnam, Sara Muñoz-González, Liani Coronado, María Teresa Frías, Carmen Laura Perera, Llilianne Ganges, Nicolas Ruggli

**Affiliations:** 1 Division of Virology, Institute of Virology and Immunology IVI, 3147 Mittelhäusern, Switzerland; yoandri11@yahoo.es (Y.H.); matthias.liniger@ivi.admin.ch (M.L.); obdulio.garcia-nicolas@ivi.admin.ch (O.G.-N.); markus.gerber@ivi.admin.ch (M.G.);; 2 Department of Infectious Diseases and Pathobiology (DIP), University of Bern, 3012 Bern, Switzerland; 3 Graduate School for Cellular and Biomedical Sciences (GCB), University of Bern, 3012 Bern, Switzerland; 4 Centro Nacional de Sanidad Agropecuaria (CENSA), San José de las Lajas 32700, Cuba; friaslepoureau@gmail.com (M.T.F.); claura@censa.edu.cu (C.L.P.); 5 WOAH Reference Laboratory for Classical Swine Fever, IRTA-CReSA, 08193 Barcelona, Spain; s.munoz@vidium-solutions.com (S.M.-G.); liani.coronado@irta.cat (L.C.); llilianne.ganges@irta.cat (L.G.); 6 Unitat Mixta d’Investigació IRTA-UAB en Sanitat Animal, Centre de Recerca en Sanitat Animal (CReSA), 08193 Barcelona, Spain; 7 IRTA, Programa de Sanitat Animal, Centre de Recerca en Sanitat Animal (CReSA), 08193 Barcelona, Spain

**Keywords:** CSFV, subgenotype 1.4, virulence, pathogenicity, reverse genetics, E2, NS5A, NS5B

## Abstract

Classical swine fever (CSF) has been eradicated from Western and Central Europe but remains endemic in parts of Central and South America, Asia, and the Caribbean. CSF virus (CSFV) has been endemic in Cuba since 1993, most likely following an escape of the highly virulent Margarita/1958 strain. In recent years, chronic and persistent infections with low-virulent CSFV have been observed. Amino acid substitutions located in immunodominant epitopes of the envelope glycoprotein E2 of the attenuated isolates were attributed to positive selection due to suboptimal vaccination and control. To obtain a complete picture of the mutations involved in attenuation, we applied forward and reverse genetics using the evolutionary-related low-virulent CSFV/Pinar del Rio (CSF1058)/2010 (PdR) and highly virulent Margarita/1958 isolates. Sequence comparison of the two viruses recovered from experimental infections in pigs revealed 40 amino acid differences. Interestingly, the amino acid substitutions clustered in E2 and the NS5A and NS5B proteins. A long poly-uridine sequence was identified previously in the 3′ untranslated region (UTR) of PdR. We constructed functional cDNA clones of the PdR and Margarita strains and generated eight recombinant viruses by introducing single or multiple gene fragments from Margarita into the PdR backbone. All chimeric viruses had comparable replication characteristics in porcine monocyte-derived macrophages. Recombinant PdR viruses carrying either E2 or NS5A/NS5B of Margarita, with 36 or 5 uridines in the 3′UTR, remained low virulent in 3-month-old pigs. The combination of these elements recovered the high-virulent Margarita phenotype. These results show that CSFV evolution towards attenuated variants in the field involved mutations in both structural and non-structural proteins and the UTRs, which act synergistically to determine virulence.

## 1. Introduction

Classical swine fever (CSF) is a highly contagious viral disease of domestic pigs and wild boar caused by CSF virus (CSFV). The virus has been eradicated from Western and Central Europe, the United States, Canada, Australia, and New Zealand, but remains endemic in certain Asian countries and Central and South America where it causes serious damage [1,2,3]. There is a constant risk of CSFV re-emergence worldwide, as witnessed by the outbreaks of the past decade in Ecuador [4], Brazil [5], Colombia [6], Japan, and North Vietnam [7,8].

CSFV belongs to the *Pestivirus suis* species (former Pestivirus C) of the genus *Pestivirus* within the *Flaviviridae* family (https://www.ncbi.nlm.nih.gov/taxonomy, accessed on 13 January 2024). The virion is 40 to 50 nm in size and is composed of a lipid envelope carrying the proteins E^rns^, E1, and E2 and a capsid composed of the C protein, and contains a positive-sense RNA genome of 12.3 kilobases. The CSFV RNA encodes a single large open reading frame (ORF) flanked by 5′ and 3′ untranslated regions (UTR) of typically 373 and 228 nucleotides, respectively. The 5′UTR folds to an internal ribosomal entry site (IRES) structure that mediates translation of the ORF to a large polyprotein of 3898 amino acids that is processed co- and post-translationally by the viral proteases N^pro^, NS2, and NS3 and by cellular signal peptidases into the twelve proteins: N^pro^, C, E^rns^, E1, E2, p7, NS2, NS3, NS4A, NS4B, NS5A, and NS5B (for review, see [9]). The nonstructural proteins NS3 to NS5B are the minimal proteins necessary for replication of the viral RNA, while the NS2 protease orchestrates NS2/NS3 processing [10,11]. Temporally regulated NS2/NS3 processing is crucial for the CSFV life cycle since the NS23 precursor protein is required for infectious particle formation [12]. NS5A is phosphorylated and regulates RNA replication by interacting with the RNA-dependent RNA polymerase (RdRp) NS5B and the 3′UTR (reviewed in [9]).

There is high genetic diversity among the CSFV isolates worldwide. Phylogenetic analyses are based mostly on partial genome sequencing from the 5′UTR to the E2 gene, with the E2 glycoprotein representing the driving force of CSFV evolution. Through this, CSFV has been classified into three genotypes, 1, 2, and 3, with up to seven subgenotypes for genotypes 1 and 2 and four subgenotypes for genotype 3 [1,13]. A recent study that compared 203 full-length sequences from CSFV isolated worldwide over 40 years proposes a classification involving two groups, with subgenotypes from the genotypes 1 and 3 in group I and five subgenotypes 2 in group II [14]. This suggests that several genomic regions are subject to selective pressure during virus evolution. The latter analysis identified 10 recombination events as an additional factor that shapes CSFV evolution.

The genetic diversity of CSFV reflects the broad range of virulence observed among the different isolates over the past decades. With increasing diversity, some CSFVs have evolved towards lower virulence. Accordingly, acute hemorrhagic fever with high lethality described mostly in the 1950s to 1990s for infections with highly virulent genotype 1 isolates became less frequent as CSFV evolved [13]. Genotype 2 and 3 isolates are mostly, but not exclusively of moderate virulence, inducing an acute to subacute febrile disease from which animals can recover or remain chronically infected until they eventually die. In countries like Cuba, where the disease became endemic due essentially to suboptimal disease control, the virus became low-virulent, leading to an increasing number of clinically inapparent infections favoring susceptibility to opportunistic pathogens [1,15,16]. The severity of CSF is also dependent on host factors such as genetic background, immune status and age [17,18]. Notably, age-related differences were observed experimentally with 5-day-old versus 3-week-old pigs [19]. Moreover, infections of newborn piglets with low-virulent strains can result in persistent infections with high viral load and delayed or lack of seroconversion [20]. Therefore, the definition of virulence must be linked to the particular type of pig used. Under defined host conditions—differing between studies—viral genetic determinants of CSFV virulence have been identified in nearly every gene (reviewed in [1,21]). Most CSFV virulence determinants have been defined through loss-of-function experiments [21]. Typically, mutations are applied to a high-virulent backbone by knocking out structural or functional domains or by swapping gene elements from unrelated low-virulent CSFV. Only a few studies rely on gain of function [1]. Despite numerous efforts to identify virulence determinants, no universal virulence marker has been found for CSFV yet. This underlines the complexity of studying CSFV virulence factors.

To reduce this complexity and to study virulence determinants in a more controlled context, we sought to exploit two evolutionary-related high- and low-virulent CSFV. For this, we selected the highly virulent strain CSFV/Margarita/1958 (Margarita) and the isolate CSFV/Pinar del Rio (CSF1058)/2010 (PdR) that evolved naturally to low virulence on the island of Cuba over two decades [16]. The Margarita strain used here is a virus batch derived from the original virulent CSFV/Margarita/1958 that re-emerged in Cuba in 1993 [22,23]. Since then, it has spread throughout the western part of the island [23] and is the most probable ancestor of the low-virulent PdR isolated in 2010 [16]. Phylogenetic analyses based on nucleotide sequences from the 5′UTR to the E2 gene (3361 nucleotides) revealed that all CSFV isolates from Cuba belong to a unique subgenotype 1.4 that is not found anywhere else [24]. Thus, the introduction of CSFV from outside Cuba between 1993 and 2010 is unlikely, which supports the evolutionary relationship between the two isolates selected here.

To identify the genetic changes related to the loss of virulence of PdR during evolution in the field, we first determined the complete nucleotide sequence of PdR (GenBank accession number KX576461) and compared it with partial sequences of the parental Margarita strain. This revealed seven amino acids substitutions in E2 and a unique poly-uridine (poly-U) sequence of an average of 36 nucleotides in the 3′UTR, where Margarita and all other known CSFV have five uridines [25]. With reverse genetics, we demonstrated that the poly-U sequence contributes to the low virulence of PdR in piglets, since the removal of the poly-U sequence from vPdR-36U—a cDNA-derived virus corresponding to the PdR field isolate—resulted in a gain of virulence of vPdR-5U in 5-day-old piglets, along with reduced immune activation [26].

In the present study, we determined the complete genome sequence of the Margarita strain and identified all mutations acquired by PdR during evolution in the field. With a functional Margarita cDNA clone and a gain-of-function strategy based on genome elements transferred from Margarita into the PdR backbone, we identified the virulence determinants of Margarita in 3-month-old large white specific pathogen-free (SPF) pigs.

## 2. Materials and Methods

### 2.1. Cells and Viruses

The original isolates and the cDNA-derived parental and chimeric viruses (see below) were amplified and titrated in the porcine aortic endothelial cell line PEDSV.15, kindly provided by J. Seebach, University of Geneva, Switzerland [27]. The PEDSV.15 cells were propagated in Dulbecco’s modified Eagle medium (DMEM, Gibco, Thermo Fisher Scientific, Waltham, MA, USA) supplemented with sodium pyruvate, non-essential amino acids, 7% heat-inactivated horse serum (HS), and 2% porcine serum (Gibco, Thermo Fisher Scientific) at 37 °C and 5% CO_2_. The porcine kidney cell line SK-6 was propagated in Earle’s minimal essential medium (Thermo Fisher Scientific) supplemented with 7% heat-inactivated HS. Porcine monocyte-derived macrophages (MDMs) were prepared from porcine blood of 6- to 12-month-old large white SPF pigs from the breeding facility of the Institute of Virology and Immunology IVI in Mittelhäusern, Switzerland, and differentiated using macrophage colony-stimulating factor (M-CSF) essentially as described elsewhere [28]. Briefly, peripheral blood mononuclear cells were isolated from Alsever’s blood using Ficoll–Paque Plus density centrifugation (GE Healthcare, Chicago, IL, USA). Monocytes were then enriched by positive selection for CD172a with the monoclonal antibody clone 74-22-15A (hybridoma kindly provided by A. Saalmüller, Veterinary University of Vienna, Austria) using a magnetic cell sorting system (MACS) with LS columns (Miltenyi Biotec GmbH, Bergisch Gladbach, Germany). The enriched monocytes were seeded at a density of 5 × 10^5^ cells per milliliter in DMEM without phenol red, supplemented with GlutaMax (Thermo Fisher Scientific), 10% pestivirus-free fetal bovine serum (FBS), and recombinant porcine M-CSF (20 U/mL) and cultured at 39 °C with 5% CO_2_ for 72 h for differentiation to macrophages.

The low-virulent CSFV/Pinar del Rio (CSF1058)/2010 (PdR) isolate was recovered from RNA from the original field isolate, as described earlier [20,25]. The highly virulent CSFV Margarita strain was isolated in Cuba in 1958 [23]. In the present study, serum collected from a pig (pig #12) 12 days after infection with the Margarita strain at the Unitat mixta d’Investigació IRTA-UAB en Sanitat Animal, Centre de Recerca en Sanitat Animal (CReSA), Barcelona, Spain [29], was shipped to the IVI, Mittelhäusern, Switzerland.

### 2.2. Margarita Genome Sequence

The complete genome sequence of the Margarita strain was determined from RNA isolated from the pig serum obtained from IRTA-UAB/CReSA described above, using TRIzol reagent (Thermo Fisher Scientific). The genome was amplified by overlapping reverse transcription (RT) and PCR. RT was performed with Superscript III reverse transcriptase (Thermo Fisher Scientific), and Phusion Hot Start II High-Fidelity DNA Polymerase (Thermo Fisher Scientific) was used for PCR. Six overlapping DNA fragments were designed to cover the complete genome except the 5′ and 3′UTRs (see Table S1 for primer sequences). The amplicons from four independent RT-PCR for each of the fragments were inserted into the TOPO TA cloning vector (TOPO TA Cloning Kit for Sequencing, Thermo Fisher Scientific). The 5′ and 3′ ends of the viral genome were determined using the 5′ and 3′ RACE System for Rapid Amplification of cDNA Ends (Thermo Fisher Scientific). The plasmid inserts were sequenced bi-directionally with forward and reverse primers using standard dideoxy-chain terminator sequencing with the BigDye Terminator v3.1 Cycle Sequencing Kit (Thermo Fisher Scientific) and an Applied Biosystems 3130 Genetic Analyzer (Thermo Fisher Scientific) to determine the consensus sequence (GenBank accession number OR997840).

### 2.3. Phylogenetic Analyses

The evolutionary history was inferred by using the maximum likelihood method and the general time reversible model, using the MEGA11 software package [30,31,32]. Initial trees for the heuristic search were obtained automatically by applying Neighbor-Join and BioNJ algorithms to a matrix of pairwise distances estimated using the Maximum Composite Likelihood approach and then selecting the topology with superior log likelihood values. A discrete Gamma distribution was used to model evolutionary rate differences among sites (5 categories [+G, parameter = 2.3302]). The rate variation model allowed for some sites to be evolutionarily invariable ([+I], 47.14% sites). The analysis involved 25 nucleotide sequences of complete CSFV genomes representative of the major CSFV subgenotypes (Table S2). The codon positions included were 1st+2nd+3rd+Noncoding. There were 12,351 positions in the final dataset.

### 2.4. Construction of cDNA Clones

To construct a functional cDNA clone of the Margarita isolate, the low copy number plasmid pACNR1180 [33] was modified to include a polylinker consisting of unique *Not*I, *Sal*I, *Cla*I, *Nsi*I, *Hin*dIII, *Spe*I, *Srf*I, and *Psp*XI restriction sites. The Margarita genome was amplified by RT-PCR from viral RNA extracted from the lysate of PEDSV.15 cells infected with the pig serum (pig #12) described above. The 5′ and 3′ halves of the genome were assembled in two separate constructs termed pMarga-5′h and pMarga-3′h using standard cloning procedures. Similar to the pACNR1180-based full-length cDNA clone of the PdR CSFV isolate [26], pMarga-5′h consists of a T7 polymerase promoter sequence for in vitro transcription of the viral genome. The unique *Spe*I site within the NS4B gene was used to generate the full-length pMargarita cDNA clone used to derive vMargarita (see below). The sequences of pMarga-5′h, pMarga-3′h, and pMargarita were verified by Sanger sequencing and were identical to the Margarita genome sequence (GenBank accession number OR997840). The constructions of the cDNA clones to derive the vPdR-36U virus, corresponding to the wild-type PdR strain, and the vPdR-5U virus lacking the large poly-U sequence were described previously [26]. To construct chimeric PdR viruses expressing the Margarita E2 glycoprotein, a synthetic PdR E2 gene cassette encoding the seven Margarita-specific E2 amino acids identified previously [25] was designed (GenScript, Leiden, The Netherlands) and used to substitute PdR E2 sequences in pPdR-5′h, resulting in pPdR-E2-5′h using standard cloning techniques. Full-length pPdR-E2-36U and pPdR-E2-5U were then assembled from pPdR-E2-5′h and pPdR-36U-3′h or pPdR-E2-5′h and pPdR-5U-3′h, respectively, using the conserved *Spe*I junction sites. The plasmids pPdR-NS5AB-5U and pPdR-E2-NS5AB-5U were obtained through substituting the NS5AB coding sequence of pPdR-5U-3′h with Margarita NS5AB, using a PCR-based strategy, resulting in pPdR-NS5AB-5U-3′h. Full-length assembly was performed with the corresponding 5′ half clones, pPdR-5′h and pPdR-E2-5′h, respectively. PCR-based methods were applied to generate the two half clones pPdR-E2-5′UTR-5′h and pPdR-NS5AB-3′UTR-3′h required for the assembly of vPdR-5′UTR-E2-NS5AB-3′UTR. The cDNA clone pPdR-E2-p7-NS234AB5AB-5U was constructed after combining the two half clones pPdR-E2-p7-NS-5′h and pPdR-NS-5′h that were generated using PCR-based and standard cloning methods. The PCR and fusion-PCR reactions were performed using Phusion Hot Start II DNA Polymerase (Thermo Fisher Scientific) with commercially obtained oligonucleotides (Microsynth, Balgach, Switzerland). All constructs generated were verified by Sanger DNA sequencing.

The bicistronic firefly luciferase replicon plasmids were constructed by replacing the N^pro^-E1 gene cassette of the recombinant vPdR/Margarita chimera described above with the corresponding N^pro^-Luc-IRES-C-E1 gene cassette from the bicistronic pA187-N^pro^-Luc-IRES-C-delE^rns^ replicon construct [34] using standard cloning procedures. Detailed information can be obtained upon request.

### 2.5. Virus Rescue from cDNA Clones

The cDNA-derived viruses were rescued as previously described [26]. The nomenclature of the rescued viruses corresponds to the respective plasmid name beginning with a v instead of a p (e.g., vMargarita was rescued from the cDNA clone pMargarita). Briefly, the plasmids were linearized with *Srf*I, and RNA was produced using the MEGAscript T7 kit (Thermo Fisher Scientific). The in vitro-transcribed RNA was treated with DNase and purified using S-400 HR columns (GE Healthcare Life Sciences, Milwaukee, WI, USA). Then, 8 × 10^6^ PEDSV.15 cells were mixed with 1 μg of RNA in 0.4 mL of ice-cold phosphate-buffered saline (PBS). Electroporations were performed using an ECM^®^ 830 electroporator (BTX, Holliston, MA, USA) with the following settings: 980 V, two pulses of 100 µs pulse length at a 0.1 s interval. The cultures were incubated in 75 cm^2^ flasks at 37 °C and 5% CO_2_ for three days. The specific infectivity of individual RNA transcripts was verified with an infectious center assay, performed as previously described [35]. The identity of the rescued viruses was determined through sequencing of specific RT-PCR fragments.

### 2.6. Virus Amplification and Titration

Virus stocks were produced in PEDSV.15 cells infected at a multiplicity of infection (MOI) of 0.1 50% tissue culture infective dose (TCID_50_)/cell. The virus was harvested at 65 to 72 h after infection by means of one freeze–thaw cycle. Viral titers were determined by endpoint dilution in PEDSV.15 cells or MDMs. CSFV was detected by immunoperoxidase staining of E2 with the HC/TC-26 monoclonal anti-E2 hybridoma supernatant as described elsewhere [36]. The titers were calculated using the Reed and Muench formula [37] and expressed in TCID_50_ per mL.

### 2.7. Virus Replication in Cell Culture

For virus replication kinetics in porcine MDMs, 0.5 × 10^6^ cells were infected in triplicate at an MOI of 0.02 or 0.001 TCID_50_/cell (according to titers from MDMs) in 24-well plates using DMEM with GlutaMax and 3% FBS (one plate per timepoint). The inoculum was removed after 1 h at 37 °C, and the cells were washed once with serum-free medium followed by incubation in DMEM with GlutaMax and 3% FBS at 39 °C and 5% CO_2_. The virus was harvested at different times post-infection by means of one freeze–thaw cycle, and the supernatant was cleared from cell debris by low-speed centrifugation at 3000× *g* for 10 min at 4 °C. The virus titer was determined by endpoint dilution in SK-6 cells as described above.

### 2.8. Experimental Infection of Pigs

All parental field strains and cDNA-derived parental and chimeric CSFV were evaluated for their virulence phenotype in 3-month-old (11- to 13-week-old) male (castrated) and female large white SPF pigs obtained from the IVI breeding facility. The animals were allocated randomly to groups of *n* = 5 pigs, with blocking for an equivalent distribution of litter and sex. The animals were infected via the oronasal route with 10^5^ TCID_50_ per pig in 5 mL MEM supplemented with 2% HS (2.5 mL intranasally and 2.5 mL orally). Body temperature and clinical score [38] were monitored daily in a blind manner by a veterinarian. Blood was sampled at two- to three-day intervals for monitoring viremia. GraphPad Prism 8 software was used for statistical analyses.

### 2.9. Viral RNA Extraction and RT-qPCR

The CSFV RNA was isolated from sera using the NucleoMag VET kit (Macherey-Nagel, Düren, Germany) and the Kingfisher Flex extraction robot (Thermo Fisher Scientific) according to the manufacturer’s instructions. Viral RNA was quantified by RT-qPCR using the AgPath-ID One-Step RT-PCR Kit (Thermo Fisher Scientific) with the forward oligonucleotide CSFV-PdR-F (5′-ATG CCC ACA GTA GGA CTA GCA-3′), the reverse oligonucleotide CSFV-PdR-R (5′-CTA CTG ACG ACT GCC CTG TAC-3′), and the probe CSFV-PdR-P (5′-FAM-TGG CGA GCT CCC TGG GTG GTC TAA GC-BHQ1-3′) (Microsynth, Balgach, Switzerland) (adapted from [39]).

### 2.10. Replicon-Mediated Firefly Luciferase Activity

For the measurement of CSFV replicon-mediated firefly luciferase activity, 3 × 10^6^ PEDVS.15 cells were electroporated with 1 μg of replicon T7-RNA in 0.4 mL of ice-cold phosphate-buffered saline (PBS). Electroporations were performed using an ECM^®^ 830 electroporator (BTX, Holliston, MA, USA) with the following settings: 980V and two pulses of 100 µs pulse length at a 0.1 s interval. After 16 and 24 h, the cells were lysed through the addition of 1× Firefly Luciferase Lysis Buffer (Biotium, Fremont, CA, USA), and cell extracts were assayed for firefly luciferase activity (Firefly Luciferase Assay Kit 2.0, Biotium) using white 96-well microplates (Lumitrac, Greiner Bio-One, Kremsmünster, Austria) and a GloMax^®^ plate reader (Promega, Madison, WI, USA). To normalize firefly luciferase activity to the efficiency of replicon T7-RNA transfection, replica cultures of electroporated PEDSV.15 cells were subjected to E2 immunostaining and flow cytometry quantification.

### 2.11. Ethics Statement

The experimental infection of the pigs was carried out in the BSL3-Ag containment facilities of the IVI (Mittelhäusern, Switzerland), in strict compliance with the Swiss animal protection law (TSchG SR 455; TSchV SR 455.1; TVV SR 455.163) and good animal practice as defined by European Union regulations. The committee on animal experiments of the canton of Bern, Switzerland, reviewed the experiments. The cantonal veterinary authority (Amt für Landwirtschaft und Natur LANAT, Veterinärdienst VeD, Bern, Switzerland) approved the study under the authorizations BE105/15 and BE74/19. Porcine blood was collected from SPF-pigs from the IVI breeding facility under the licenses BE131/17 (approval date 12 January 2018) and BE127/2020 (approval date 2 March 2021).

## 3. Results

### 3.1. The Amino Acid Mutations Acquired by PdR during Natural Evolution and Attenuation Are Clustering in E2, NS5A, and NS5B

So far, partial sequencing data have revealed differences between CSFV Margarita and PdR in E2 and the 3′UTR [25]. Here, we determined the complete nucleotide sequence of the highly virulent Margarita strain (CSFV Margarita/1958, GenBank accession number OR997840). The genome consists of 12,298 nucleotides, with a 5′UTR of 373 nucleotides, a 3′UTR of 228 nucleotides, and an ORF encoding 3898 amino acids, as with most CSFV strains.

**Figure 1:**
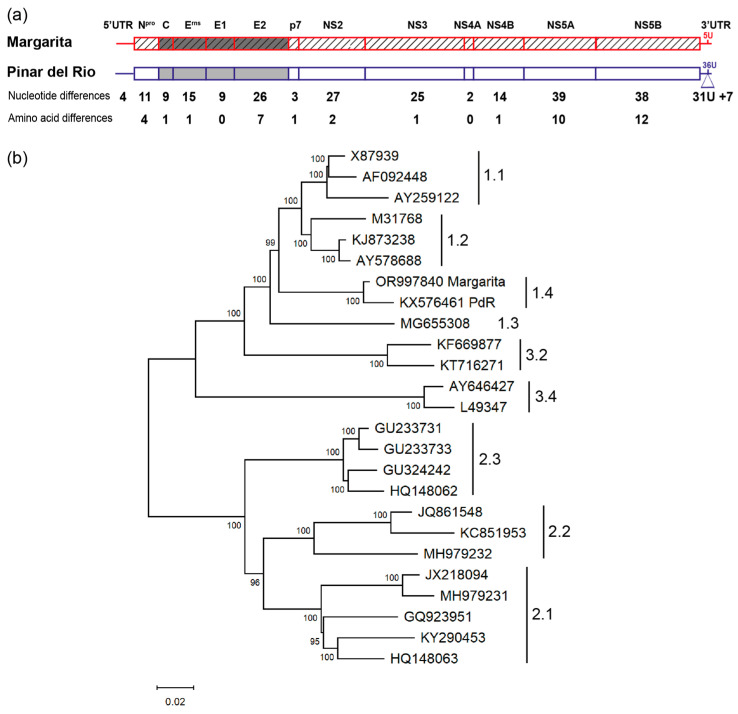
Genetic and phylogenetic analysis of the CSFV subgenotype 1.4 isolates Margarita and PdR. (**a**) Schematic representation of the genomes of CSFV Margarita (GenBank accession number OR997840) and PdR (GenBank accession number KX576461). The number of nucleotide and amino acid differences are indicated for the 5′ and 3′UTRs and the viral genes and the corresponding proteins, respectively. The poly-U insertion (31U) at the 3′UTR of PdR is shown with a triangle. (**b**) Phylogenetic tree of selected full-length CSFV genome sequences from subgenotypes 1.1, 1.2, 1.3, 1.4, 2.1, 2.2, 2.3, 3.2, and 3.4 (Table S2). The viruses are named with their GenBank accession number. The evolutionary analyses were conducted with MEGA11 [32]. The tree with the highest log likelihood (−73,547.97) is shown. The percentage of trees in which the associated taxa clustered together is shown next to the branches. The tree is drawn to scale, with branch lengths measured in the number of substitutions per site (scale bar = 0.02 substitutions/site).

Nucleotide sequence comparison of Margarita and PdR revealed a total of 260 differences, 31 of which formed a poly-U sequence of 36 uridines (36U) together with the standard 5 uridines found at nucleotides 12,225 to 12,229 of Margarita as described before [25] (Figure 1a). The 3′UTR had seven additional differences, one of which was a uridine deletion in PdR, corresponding to one of the four uridines found at positions 12,139 to 12,142 of the Margarita genome. Four differences were found in the 5′UTR. One was an adenine insertion in PdR resulting in six adenines instead of the standard five adenines found in Margarita and most CSFV genomes at positions 350–354. The remaining 218 nucleotide differences were evenly distributed along the ORF with a high proportion of synonymous substitutions. This contrasted with the amino acid substitutions clustering in E2, NS5A and NS5B: among the 40 amino acid differences found between Margarita and PdR, seven were in E2 (as described earlier [25]), ten were in NS5A, and twelve were in NS5B (Figure 1a and Table 1). No amino acid difference was found in E1 nor in NS4A despite nine and two nucleotide differences, respectively. The other viral proteins had one (C, E^rns^, p7, NS3, and NS4B), two (NS2), or four amino acid mutations (N^pro^), some of which were conservative. This suggests that E2, NS5A and NS5B adapted their protein sequences during attenuation in the field, while there was a functional selective pressure on the other genes to conserve the viral protein sequences. From this comparison, we hypothesized that E2, NS5A, NS5B, and the poly-U sequence were the key players of PdR attenuation and consequently Margarita virulence.

A phylogenetic analysis in which the full-length sequences of Margarita and PdR were compared with 23 other full-length CSFV genomes representative of the three genotypes and the major subgenotypes (Table S2) confirmed the classification of Margarita and PdR in subgenotype 1.4 found solely in Cuba (Figure 1b). The latter supports the natural evolutionary relationship of these two viruses.

### 3.2. Functional Validation of cDNA-Derived vMargarita and vPdR-36U In Vitro and In Vivo

**Figure 2:**
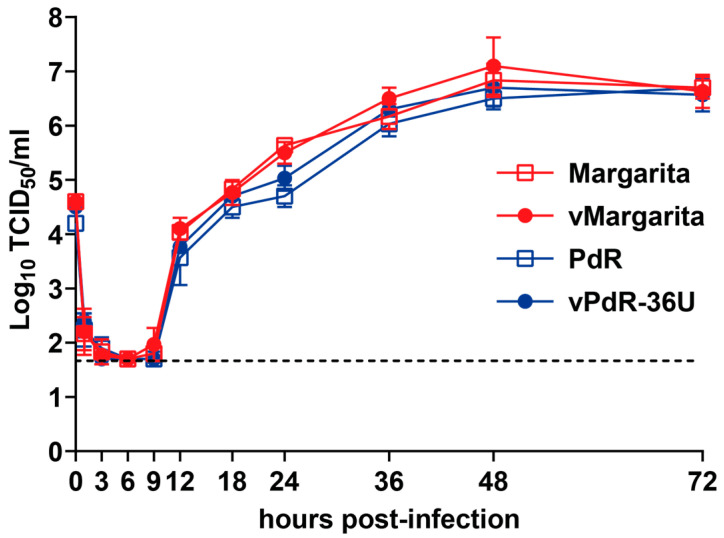
Replication kinetics of cDNA-derived versus parental CSFV Margarita and PdR in porcine MDMs. MDMs were infected in triplicate with the CSFV field isolates Margarita or PdR or with the respective cDNA-derived vMargarita or vPdR-36U viruses at an MOI of 0.02 TCID_50_/cell based on titers obtained in MDMs. At the indicated hours post-infection, the viruses were harvested by means of one freeze–thaw cycle and the infectious titers were determined in SK-6 cells through endpoint dilution. Each dot represents the mean titer from three replica infections, with error bars showing standard deviations (SDs). The dashed line shows the limit of detection in the titration assay (1.7 log_10_ TCID_50_/mL).

We applied state-of-the-art reverse genetics to verify the above hypothesis and demonstrate which genes or sequence elements determine the virulence phenotype of the Margarita isolate. For this, we first assembled a full-length cDNA clone of the Margarita strain with the consensus sequence determined above, as we did before with the PdR isolate [25]. We rescued the vMargarita virus from PEDSV.15 cells transfected with in vitro transcripts and verified its nucleotide sequence. A potential drawback of using cDNA-derived viruses is that they carry a reconstructed consensus sequence that does not necessarily represent the genome sequences resulting in the phenotype of the heterogenous parental virus stock [45]. Therefore, it was essential to validate the phenotypes of the cDNA-derived vMargarita and vPdR-36U in comparison with their respective parental field isolates Margarita and PdR in primary porcine MDMs and SPF pigs. The cDNA-derived viruses were indistinguishable from their respective parental viruses in terms of replication kinetics in MDMs (Figure 2). Importantly, there was no significant difference between the high- and low-virulent viruses in the cell culture (Figure 2).

**Figure 3:**
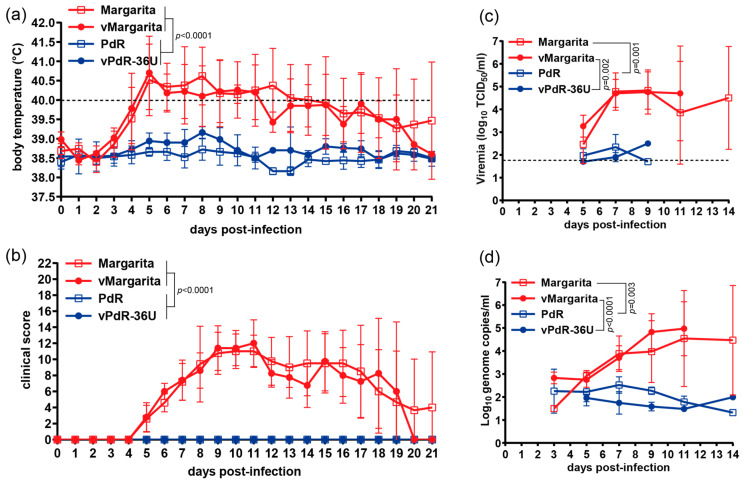
Phenotype of cDNA-derived versus parental CSFV Margarita and PdR in SPF pigs. Groups of 3-month-old large white SPF pigs (*n* = 5/group) were infected via the oronasal route with 10^5^ TCID_50_/pig of the field isolates Margarita or PdR or the respective cDNA-derived viruses vMargarita or vPdR-36U, as described in the Materials and Methods. Body temperature (**a**), clinical score (**b**), infectious virus titer (**c**), and number of genome copies (**d**) per milliliter of serum were monitored daily or every 2 to 3 days as indicated. Body temperatures above 40 °C (dashed line) were considered as fever (**a**). The dashed line in (**c**) shows the limit of detection in the titration assay (1.7 log_10_ TCID_50_/mL). Each dot represents the mean value from five pigs, with error bars showing the SDs. Differences between groups were analyzed by comparing the area under the curve (AUC) using ordinary one-way ANOVA with Tukey’s post hoc test. The adjusted *p*-values are indicated where the differences are significant.

This contrasted with replication and pathogenicity in pigs (Figure 3). Margarita and vMargarita induced prolonged fever and severe clinical signs in 3-month-old SPF pigs (*n* = 5 each, Figure 3a,b). These viruses reached on average 3 log_10_ higher viral titers and genome copies in the blood for several days compared with PdR and vPdR-36U (*n* = 5 each, Figure 3c,d). The latter did not cause any elevated body temperatures nor disease symptoms in the pigs (Figure 3a,b). The differences were highly significant when the area under the curve (AUC) was compared (*p_adj_* < 0.0001 for body temperatures and clinical scores and *p_adj_* < 0.003 for virus titers and log genome copies in the blood). Importantly, cDNA-derived vMargarita and vPdR-36U were indistinguishable phenotypically from their respective parental Margarita and PdR field isolates (AUC *p_adj_* > 0.9 for viremia and *p_adj_* > 0.3 and 0.9 for body temperatures and clinical scores, respectively). These data validate the use of the two cDNA backbones for the construction of recombinant chimeric viruses and reverse genetic analyses.

### 3.3. Neither E2 of Margarita nor 5 Uridines in the 3′UTR of the PdR Backbone Can Restore Virulence in 3-Month-Old SPF Pigs

**Figure 4:**
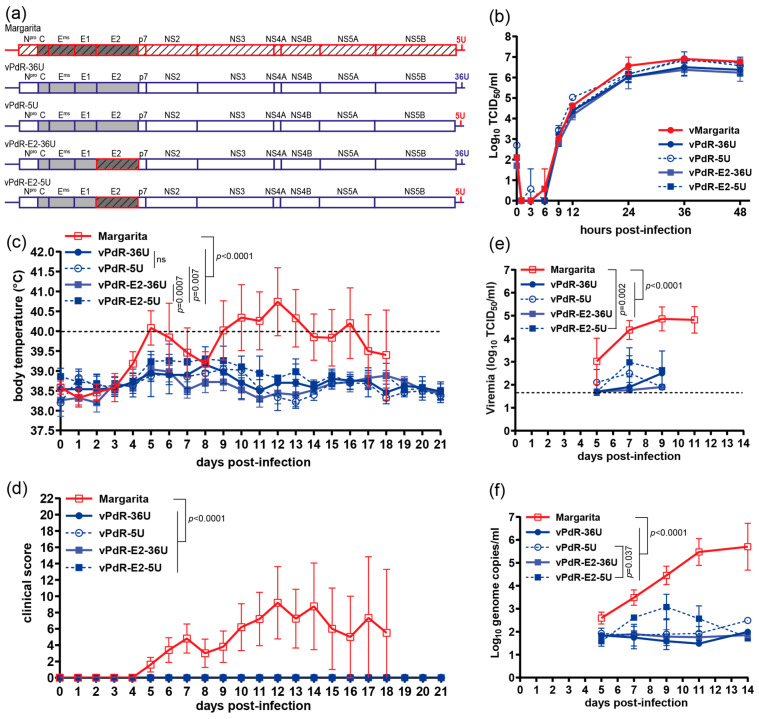
Effect of 5 versus 36 uridines of the 3′UTR and E2 from Margarita in PdR on replication in MDMs and virulence in SPF pigs. (**a**) Schematic representation of the chimeric CSFV genomes with sequences from the Margarita genome (red) in the PdR genome (blue), i.e., with either 5 instead of 36 uridines in the 3′UTR (vPdR-5U) or E2 from Margarita (vPdR-E2-36U) or the combination of the two features (vPdR-E2-5U) in the PdR backbone. (**b**) MDMs were infected in triplicate with the respective cDNA-derived viruses at an MOI of 0.001 TCID_50_/cell based on titers obtained in MDMs. At the indicated hours post-infection, viruses were harvested by means of one freeze–thaw cycle and the infectious titers were determined in SK-6 cells through endpoint dilution. Each dot represents the mean titer from three replica infections, with error bars showing the SDs. (**c**–**f**) Groups of 3-month-old large white SPF pigs (*n* = 5/group) were infected via the oronasal route with 10^5^ TCID_50_ of the Margarita field isolate or the cDNA-derived viruses as indicated. Body temperature (**c**), clinical score (**d**), infectious virus titers (**e**), and number of genome copies (**f**) per milliliter of serum were monitored daily, every 2 to 3 days as indicated. Body temperatures above 40 °C (dashed line) were considered as fever (**c**). The dashed line in (**e**) shows the limit of detection in the titration assay (1.7 log_10_ TCID_50_/mL). Each dot represents the mean from five pigs, with error bars showing the SDs. Differences between groups were analyzed by comparing the area under the curve (AUC) using ordinary one-way ANOVA with Tukey’s post hoc test. The adjusted *p*-values are indicated where the differences are significant.

Having functionally validated the cDNA clones, we successively introduced individual genes and/or UTR sequences from vMargarita into the vPdR-36U backbone and evaluated the recombinant viruses for replication in porcine MDMs and for a potential gain of virulence in 3-month-old SPF pigs. First, we compared vPdR-5U shown previously to have gained virulence in newborn piglets [26], with vPdR-36U and with PdR backbones encoding E2 of Margarita (vPdR-E2-36U) or carrying both, the Margarita E2 amino acid sequence and 5 instead of 36 uridines in the 3′UTR (vPdR-E2-5U) as shown schematically in Figure 4a.

The cDNA-derived parental and chimeric viruses rescued in PEDSV.15 cells had the correct nucleotide sequences. Their growth characteristics were indistinguishable in the cell culture, as witnessed by a multistep growth curve in porcine MDMs (Figure 4b). This demonstrates that the viruses are fully functional. In 3-month-old SPF pigs (*n* = 5), no signs of disease were observed at any time after infection with any of the four PdR-derived viruses, which was significantly different from infections with the Margarita field isolate (Figure 4c,d; AUC *p_adj_* < 0.0001 for body temperatures and clinical scores, respectively). The Margarita virus was virulent as expected, with a characteristic biphasic body temperature and clinical score curve (Figure 4c and Figure 4d, respectively) as observed repeatedly (see also Figure 3). Accordingly, the viremia and number of genome copies per milliliter serum were significantly lower for any of the four PdR-derived viruses when compared with the Margarita field isolate (Figure 4e,f). Interestingly, as shown in Figure 4c, the body temperatures of pigs infected with vPdR-E2-5U were slightly but significantly more elevated when the AUC was compared with the vPdR-E2-36U (*p_adj_* = 0.0007) or vPdR-5U (*p_adj_* = 0.007) infections. Notably, there was no significant difference in body temperatures between the vPdR-36U- and vPdR-5U-infected pigs (AUC *p_adj_* = 0.4). Consistent with the body temperatures, the number of genome copies in serum was slightly higher for vPdR-E2-5U when compared with vPdR-5U (Figure 4f; AUC *p_adj_* = 0.037). For the other PdR-derived mutants the differences in viremia were not significant (Figure 4e,f). These results suggest a minor contribution of E2 to virulence, which may be enhanced synergistically in the absence of the poly-U sequence.

### 3.4. E2 and NS5A-NS5B Act Synergistically to Determine the Margarita Virulence Phenotype in 3-Month-Old Pigs

**Figure 5:**
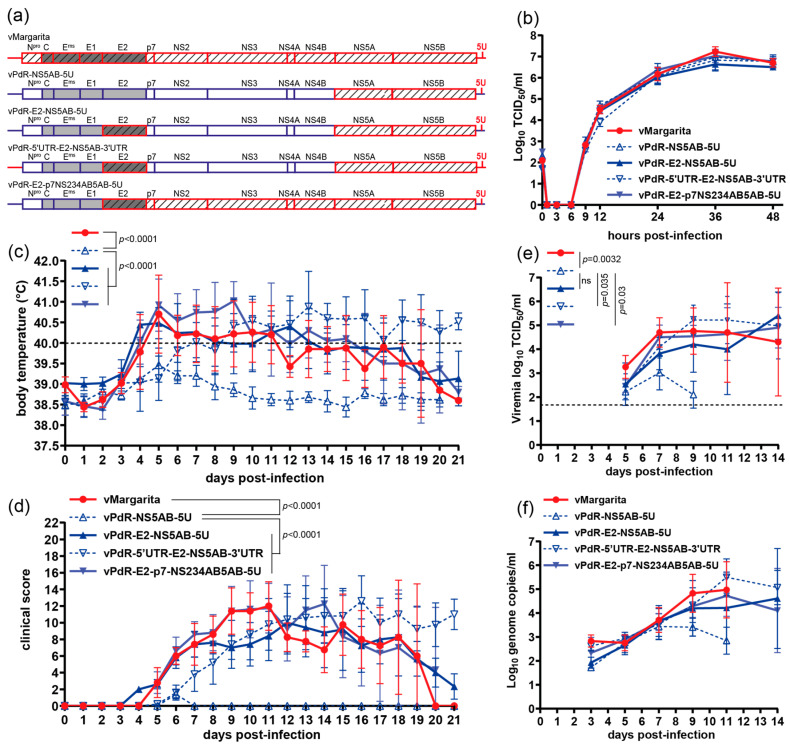
E2 and NS5AB from Margarita act synergistically in the PdR backbone to restore the Margarita virulence phenotype in pigs. (**a**) Schematic representation of the chimeric CSFV genomes with genes from vMargarita (red) in the PdR backbone (blue), with 5 uridines in the 3′UTR or the entire 5′ and 3′UTRs of Margarita. (**b**) MDMs were infected in triplicate with the respective cDNA-derived viruses at an MOI of 0.001 TCID_50_/cell based on titers obtained in MDMs. At the indicated hours post-infection, the viruses were harvested by means of one freeze–thaw cycle and the infectious titer was determined in SK-6 cells through endpoint dilution. Each dot represents the mean titer from three replica infections, with error bars showing the SDs. (**c**–**f**) Groups of 3-month-old large white SPF pigs (*n* = 5/group) were infected via the oronasal route with 10^5^ TCID_50_ of the indicated cDNA-derived viruses. Body temperature (**c**), clinical score (**d**), infectious virus titers (**e**), and number of genome copies (**f**) per milliliter of serum were monitored daily, every 2 to 3 days as indicated. Body temperatures above 40 °C (dashed line) were considered as fever (**c**). The dashed line in (**e**) shows the limit of detection in the titration assay (1.7 log_10_ TCID_50_/mL). Each dot represents the mean from five pigs, with error bars showing the SDs. Differences between groups were analyzed by comparing the area under the curve (AUC) using ordinary one-way ANOVA with Tukey’s post hoc test. The adjusted *p*-values are indicated where the differences are significant.

Next, we evaluated the potential contribution of NS5A-NS5B (NS5AB) to virulence. For this, we introduced the NS5AB cassette from Margarita into the vPdR-5U or the vPdR-E2-5U backbones, resulting in vPdR-NS5AB-5U—addressing the role of NS5AB alone—or vPdR-E2-NS5AB-5U—addressing a potential synergy between E2 and NS5AB (Figure 5a)—respectively. We also evaluated the potential role of the 5′ and 3′UTRs of Margarita by constructing vPdR-5′UTR-E2-NS5AB-3′UTR (Figure 5a). Finally, we introduced the complete E2 to NS5B polyprotein of Margarita into vPdR-5U (Figure 5a, vPdR-E2-p7-NS234AB5AB-5U), which was hypothesized to recapitulate the Margarita phenotype.

As above, we confirmed the complete nucleotide sequence of the four recombinant viruses rescued in PEDSV.15 cells and showed that their replication kinetics were indistinguishable in porcine MDMs (Figure 5b). To investigate this further in vitro, we uncoupled genome replication from virus replication involving cell-to-cell spreading by constructing bicistronic replicons corresponding to the different recombinant viruses and expressing firefly luciferase (Figure S1). None of the genome elements from Margarita conferred any replicative advantage or impairment to the PdR backbone, as demonstrated by indistinguishable luciferase activity. This showed that the chimeric genomes were fully functional, validating all recombinant viruses for further phenotypic evaluation in pigs.

In infected 3-month-old pigs, all viruses except vPdR-NS5AB-5U induced severe disease. Pigs infected with vPdR-NS5AB-5U had sub-febrile body temperatures of 39.1–40 °C on days 5 and 6 after infection, along with reduced liveliness and stiffness observed in all five animals on day 6 (Figure 5c and Figure 5d, respectively). The differences in body temperature and clinical score between vPdR-NS5AB-5U-infected pigs and pigs infected with any of the other four viruses were highly significant when the AUCs were compared (Figure 5c and Figure 5d, respectively; *p_adj_* < 0.0001 for all). The differences in viremia (Figure 5e) were less pronounced but significant when the AUC of the infectious titers of vPdR-NS5AB-5U was compared with the AUC of vMargarita (*p_adj_* = 0.0032), vPdR-5′UTR-E2-NS5AB-3′UTR (*p_adj_* = 0.035), and vPdR-E2-p7-NS234AB5AB-5U (*p_adj_* = 0.03) between days 5 and 9. The slight difference in viremia observed between vPdR-NS5AB-5U and vPdR-E2-NS5AB-5U was not significant (Figure 5e; AUC *p_adj_* = 0.23). The differences in genome copies were not significant either (Figure 5f; AUC *p_adj_* > 0.09). The other three chimeric viruses did not differ from the cDNA-derived parental vMargarita, neither in terms of body temperature (Figure 5c; AUC *p_adj_* > 0.3) nor clinical score (Figure 5d; AUC *p_adj_* > 0.9) viremia (Figure 5e; AUC *p_adj_* > 0.4), or genome copies (Figure 5f; AUC *p_adj_* > 0.7).

These latter results indicate that the RdRp NS5B, in conjunction with NS5A, contributes to virulence. But as for the seven amino acid substitutions of E2 alone, the 22 amino acid mutations of NS5AB alone are not sufficient to confer the Margarita phenotype to PdR. This was drastically different when the two domains were combined in the PdR backbone.

Altogether, the present study demonstrates with a gain-of-function approach that the virulence phenotype of CSFV Margarita is the result of a synergistic effect between the envelope protein E2 and the replicase proteins NS5A and NS5B. Conversely, one can conclude that the attenuated phenotype of PdR is the result of adaptive mutations in both, E2 and NS5AB, with a minor contribution of the poly-U insertion in the 3′UTR.

## 4. Discussion

This study uses two natural evolutionary-related high and low-virulent CSFV isolates from the island of Cuba to identify viral genetic determinants of CSFV virulence. The low-virulent CSFV PdR [25] was isolated nearly two decades after the high-virulent CSFV Margarita had re-emerged in the western part of the island in 1993 and has become endemic to the present day [15,23]. By comparing the entire nucleotide sequences of the two isolates, we identified the genetic changes accumulated during attenuation. We then applied reverse genetics for the step-by-step reconstruction of the Margarita genome in the PdR backbone and measured gain of function in vivo. With this approach, we demonstrate that the mutations related to virulence were not randomly distributed along the genome but rather clustered in E2 and in NS5A and NS5B, with a minor contribution of the 3′UTR.

A comparison of the two full-genome sequences revealed a total of 260 nucleotide and 40 amino acid differences. Of these 40 amino acid differences, 7 were located in E2 as described and discussed earlier in the context of vaccination-related selection [16,25], and 22 clustered in NS5A and NS5B. Together, these mutations represent 73% of all amino acid differences found (Figure 1a). This leaves the other viral proteins with one to four amino acid substitutions and E1 and NS4A without any changes, despite a considerable number of synonymous nucleotide substitutions accumulated in these genes. Earlier partial sequencing revealed the seven amino acid changes in E2 and a novel 36 polyuridine sequence in the 3′UTR of PdR [16,25]. The substitutions in E2 were attributed to vaccine-mediated positive selection [16]. A combination of virulence, sequencing data and in silico modeling using different related CSFV isolates from Cuba strengthened the hypothesis that the positive selection exerted on E2 following vaccination may have contributed to attenuation [40]. Positive selection has also been suggested to drive the genetic diversity of CSFV of subgenotype 2.1 in China [46]. Another study demonstrated experimentally the occurrence of vaccination-mediated positive selection through high-throughput sequencing of C-strain-vaccinated pigs challenged with the subgenotype 1.1 strain Koslov [47]. This revealed 16 nucleotide changes that were undetectable in the inoculum and the non-vaccinated, infected pigs. In this study, two nonsynonymous changes occurred in E2, one of which also occurred in PdR but in the opposite direction (S763L in Koslov after selection, L763S in PdR). The mutation found in NS5A of Koslov did not occur in PdR. Partial sequence data of the last 379 nucleotides of NS5B and the first 30 nucleotides of the 3′UTR of Margarita and PdR are a perfect match to our data, with eight nucleotide differences resulting in two amino acid substitutions within the last 124 amino acids of NS5B and two nucleotide differences at the beginning of the 3′UTR [48].

A prerequisite for the stepwise reconstruction of the Margarita genome in the PdR backbone through reverse genetics was to demonstrate that the cDNA-derived parental viruses vMargarita and vPdR-36U had the same phenotype as their respective original field isolates in terms of replication kinetics in porcine MDMs and virulence in pigs (Figure 2 and Figure 3). The course and intensity of disease and symptoms we observed experimentally with CSFV Margarita that caused the 1993–1997 epizootic [23] and cDNA-derived vMargarita were indistinguishable. Our observations with Margarita in SPF-pigs are consistent with results from experimental infections reported earlier with this isolate [48,49]. Contrasting with Margarita, the PdR isolate and the cDNA-derived vPdR-36U did not induce any detectable sign of disease in any of the SPF-pigs. This is different from the mild symptoms observed by others with PdR in the field or in experimental infections [16,48]. This discrepancy is most probably attributable to the exceptional hygiene status of the SPF pigs used in the present study, as we showed recently with African swine fever virus (ASFV) [50]. The avirulent phenotype of PdR in 3-month-old SPF pigs also differs from the infection of newborn piglets, in which PdR induced chronic disease with moderate symptoms and/or persistence [20,26]. Validation of parental and cDNA-derived CSFV was considered essential, since the phenotypes of cDNA-derived RNA viruses relate to a unique reconstructed sequence, which in our case corresponds to the consensus sequence of the parental genome. With the CSFV Koslov isolate, Fahnøe and co-workers showed that the uncloned parental virus can contain a considerable number of non-functional genomes [51]. Reconstruction of the consensus sequence in the backbone of four different non-functional cDNA genomes of the same parental virus resulted in viruses with different replication and virulence phenotypes [45]. The use of consensus cDNA sequences ignores any potential effect on virulence of a quasispecies mixture of field isolates. A former descriptive study identified higher quasispecies diversity in highly virulent CSFV as opposed to CSFV of lower virulence, leaving the question open as to whether quasispecies diversity may relate to virulence [52]. With the present data, and in agreement with the study mentioned above [45], we can conclude that the phenotype of Margarita and PdR is independent of quasispecies, since the field and cDNA-derived viruses were indistinguishable. Comparison of acute and chronic CSFV infections did not reveal any link to specific quasispecies patterns either [53].

Having validated the cDNA clones of the parental viruses, we introduced genome elements from vMargarita into the vPdR-36U backbone (Figure 4 and Figure 5), focusing on the genome regions with the majority of changes. Importantly, none of the recombinant viruses and replicons were impaired in their basic genome replication characteristics nor in their replication kinetics in primary cell culture (Figure 4b, Figure 5b and Figure S1). This was important for downstream validation of any gain of function based on different virulence phenotypes observed with the chimeric constructs in vivo.

Interestingly, the poly-U sequence of the 3′UTR does not determine the apathogenic phenotype of PdR in 3-month-old pigs, since the reduction of 36 to 5 uridines did not result in any signs of disease at any time after vPdR-5U infection (Figure 4c,d). This contrasts with previous observations with vPdR-5U versus vPdR-36U in 5-day-old piglets, in which the poly-U sequence contributed clearly to the low-virulence phenotype [26]. In this latter study, the vPdR-5U mutant replicated to significantly higher titers and resulted in higher clinical scores and lethality than the vPdR-36U, which was significantly less virulent but not completely apathogenic. This discrepancy is most probably attributable to the age difference—3-month-old versus 5-day-old pigs—as well as the exceptional SPF status of the pigs employed in the present study. Pigs from the same SPF breeding facility, as used in the present study, were significantly more resilient to a moderately virulent ASFV isolate than conventional farm pigs [50]. The 5-day-old piglets that developed severe disease with the same vPdR-5U as used here were raised under conventional conditions and were a different breed [26]. In addition, in 3-month-old pigs, we did not observe any persistence of vPdR-36U, contrasting with the persistence of PdR described in piglets infected at birth [20]. Age-related differences in disease severity were also observed when a virulent E^rns^ mutant PdR (vPdR-H_30_K-5U) was assessed in 3-week-old pigs in comparison with 5-day-old piglets [19]. Taken together, these data suggest a non-negligible contribution of age, genetic background, and hygiene status of the host to the virulence phenotype observed. This underlines that the virulence of CSFV is a relative definition that must also consider the condition of the host, as proposed elsewhere [1].

The seven amino acid substitutions in E2 acquired by PdR during the potential evolutionary process from Margarita were not sufficient to attenuate the virus in 3-month-old SPF pigs either, since vPdR-E2-36U and vPdR-36U were both avirulent and did not replicate differently in pigs (Figure 4), while a slight gain of function was observed when the poly-U sequence was removed in this context (vPdR-E2-5U). This contrasts with several studies showing that CSFV E2 on its own can determine CSFV virulence in pigs. During the reconstruction of the virulent Koslov virus from non-functional haplotypes, two amino-acid substitutions in E2 were found to be critical for virulence [45]. In another study, after eleven passages, an attenuated chimeric Shimen virus carrying the C-strain E2 partially regained virulence, which was associated with two mutations in E2 [54]. While the latter two studies rely on gain of function, other reports use attenuation of virulent viruses. Mutations in antigenic or fusion domains of E2 of the highly virulent Brescia strain attenuated the virus [55,56,57]. However, the mutated amino acid positions described in the latter studies are conserved in Margarita and PdR, as discussed previously [25]. Also, codon-deoptimization of E2, without altering the amino acid sequence nor the rest of the genome resulted in attenuation [58]. Glycosylation of E2 is also required for virulence since the removal of a single N-linked glycosylation site was sufficient to attenuate the virus [59]. Conversely, the introduction of an additional glycosylation site in E2 attenuated the virulent Shimen strain and resulted in protection [60]. Of note, none of the seven amino acid differences in E2 of Margarita versus PdR affected N-glycosylation motifs nor were they located in their vicinity.

Based on the results of slight gain of virulence with vPdR-E2-5U, we used this virus to introduce additional genome elements from vMargarita. Our findings demonstrate that NS5A and NS5B, in combination with E2, are sufficient to recapitulate the vMargarita phenotype, in contrast with the NS5A-NS5B cassette alone (Figure 5). Conversely, this implies that the mutations acquired by PdR in E2 and NS5A-NS5B were sufficient for the complete attenuation of Margarita. We did not further investigate the individual effects of NS5A or NS5B. The two proteins accumulated mutations, whereas the other non-structural proteins did not. This suggests a driving force for the co-evolution of NS5A and NS5B, which may be dissected in more detail in subsequent studies. The N-terminal half of NS5A, including the zinc-binding motif composed of residues C_34_, C_57_, C_59_, and C_84_ [42] is completely conserved between Margarita and PdR, except for the S2698R substitution (Table 1) located in the membrane anchor helix [41]. The 12 amino acid positions mutated in NS5A during the adaptation of PdR are neither required for interaction with NS5B [61] nor for IRES-dependent translation [62]. In NS5B, three substitutions are located in the novel N-terminal domain described recently [43], but they do not involve the conserved residues of this domain (Table 1). The other NS5B mutations are located mainly in the fingers and thumb domains [44]. The palm domain containing the G_3627_-D_3628_-D_3629_ motif required for the RdRp activity of NS5B [44,63] has only one conservative R3511K change. Finally, one substitution (T3892A) is located in the transmembrane anchor [44].

We did not explore the potential of the poly-U tract to reduce the virulence of vMargarita in vivo, which, based on the results obtained with vPdR-36U versus vPdR-5U, is unlikely to occur. Nevertheless, attenuation due to a poly-U sequence in the 3′UTR cannot be excluded, since the introduction of 12 uridines in the 3′UTR of the virulent Shimen backbone attenuated the virus [64]. In line with this, we have shown that the virulence of PdR with RNase-negative E^rns^ was drastically enhanced in piglets when the poly-U sequence was removed [19]. Only a few studies show synergistic effects between E2 and other viral elements for gain of virulence in pigs. For the GPE^−^ strain, mutations in E2 and the non-structural protein NS4B were necessary to restore the virulence of the parental ALD strain [65]. In another study, chimeric CSFV Shimen viruses were attenuated by replacing the E2 gene or the 3′UTR or both with corresponding sequences from the C-strain [66]. In the Shimen virus carrying E2 from the C-strain, two amino acid substitutions acquired in E2 after eleven virus passages partially restored virulence. In the chimeric virus carrying the C-strain 3′UTR, two mutations in NS5B also partially recovered virulence. Only a minor gain of virulence was observed in the double-chimeric virus after eleven passages, with mutations acquired in N^pro^ and NS5B.

## 5. Conclusions

The present study is one of few reports that formally identify virulence determinants of CSFV using gain of function in vivo. The lack of any difference in the replication characteristics of these parental and chimeric viruses in porcine MDMs indicates that virulence in pigs is the result of a complex virus–host interaction that cannot be linked to replication characteristics in cell culture, at least with the viruses and cells studied here. While we are aware that the results of this study are specifically restricted to CSFV isolates from Cuba, they demonstrate that the adaptive changes in E2, NS5A, NS5B and the 3′UTR were required to attenuate the Margarita strain towards the PdR phenotype. The involvement of different genes suggests that, besides the positive selection described previously on E2, other evolutive mechanisms may have exerted selective pressures on NS5A and NS5B. Thus, in addition to adaptive immunity-mediated selection, other mechanisms of virus–host interaction may also be involved in CSFV evolution and attenuation. Whether selection processes acted on NS5A with potential compensatory mutations in NS5B or vice-versa or on the two proteins independently, requires further investigation. Based on this and other studies that have applied gain of function, it seems that CSFV virulence is determined by synergistic effects between different viral structures rather than by a hypothetical universal CSFV virulence factor.

## Figures and Tables

**Table 1:** Amino acid differences between CSFV Margarita and PdR.

Gene	Nucleotide Position ^1^	Amino Acid Position	Margarita	PdR
N^pro^	t556g	61	N	K
	g567a	65	R	K
	g702a	110	R	Q
	g851a	160	D	N
C	g894a	174	S	N
E^rns^	a1527g	385	K	R
E2	g2654a	761	G	R ^2^
	t2661c	763	L	S ^2^
	a2711g	780	I	V ^3^
	c2927t	852	H	Y
	a3069t	899	D	V
	t3243c	957	L	S
	a3458g	1029	T	A ^2^
p7	t3664a	1097	D	E
NS2	c3794t	1141	R	W
	a4433g	1354	T	A
NS3	a6402g	2010	K	R
NS4B	g8024a	2551	V	I
NS5A	t8467a	2698	S	R ^4^
	g9248a	2959	V	M ^5^
	t9318c	2982	L	S ^5^
	t9330c	2986	L	P ^5^
	a9353c	2994	M	L ^5^
	g9369a	2999	G	D ^5^
	g9549a	3059	R	K ^6^
	a9641g	3090	T	A ^6^
	c9710a	3113	L	M ^6^
	g9759a	3129	G	E ^6^
NS5B	g10005a	3211	S	N ^7^
	a10158g	3262	H	R ^7^
	c10289t	3306	H	Y ^7^
	t10350c	3326	I	T ^8^
	a10478g	3369	T	A ^8^
	g10554a	3394	R	K ^8^
	g10905a	3511	R	K ^9^
	a11037g–a11038g	3555	K	R ^8^
	a11447g	3692	S	G ^10^
	a11648g	3759	T	A ^10^
	g11981a	3870	A	T
	a12047g	3892	T	A ^11^

^1^ Non-synonymous nucleotide differences between Margarita and PdR. Nucleotide numbering of the Margarita sequence; GenBank accession number OR997840. ^2^ Mutations within predicted CTL epitopes; they may facilitate immune escape [40]. ^3^ Mutation within a predicted B-cell epitope, without impact on antigenicity [40]. ^4^ Mutation within the NS5A membrane anchor helix [41]. ^5, 6^ Mutations within domain II^5^ and domain III^6^ of NS5A, respectively [42]. ^7^ Mutations within the N-terminal domain of NS5B [43]. ^8, 9, 10^ Mutations within the fingers^8^, palm^9^, and thumb^10^ domains of NS5B, respectively [44]. ^11^ Mutation within the transmembrane anchor of NS5B [44].

## Data Availability

The data presented in this study are available in the present article and supplementary materials and on GenBank (accession numbers KX576461 and OR997840).

## Supplementary Materials

The following supporting information can be downloaded at: https://www.mdpi.com/article/10.3390/v16010147/s1. Table S1: Oligonucleotides used to amplify six overlapping PCR fragments for the sequencing and assembly of the Margarita cDNA (5′ and 3′ ends determined by RACE, see Materials and Methods); Table S2: CSFV complete genome sequences selected for the phylogenetic analysis of Figure 1b; Figure S1: The firefly luciferase (FFL) expression from replicons of PdR (ΔE^rns^-36U) and Margarita (ΔE^rns^-5′UTR-E2-NS234AB5AB-3′UTR) and from chimera thereof in PEDSV.15 cells does not reflect the difference of virulence observed in vivo.
