# Supplementary material for: Evolutionary-Related High- and Low-Virulent Classical Swine Fever Virus Isolates Reveal Viral Determinants of Virulence
Source: Viruses. 2024 Jan 19;16(1):147. doi: 10.3390/v16010147 (PMC10820463; doi:10.3390/v16010147)
Supplement: Supplementary file 1 [file viruses-16-00147-s001.zip › viruses-2810517-supplementary.pdf]

viruses

MDPI

Article

Evolutionary-Related High- and Low-Virulent Classical Swine Fever Virus Isolates Reveal Viral Determinants of Virulence

Yoandry Hinojosa [1,2,3,4], Matthias Liniger [1,2], Obdulio García-Nicolás [1,2], Markus Gerber [1,2], Anojen Rajaratnam [1,2], Sara Muñoz-González [5,6], Liani Coronado [5,6,7], María Teresa Frías [4], Carmen Laura Perera [4], Llilianne Ganges [5,6,7] and Nicolas Ruggli [1,2,*]

1 Division of Virology, Institute of Virology and Immunology IVI, 3147 Mittelhäusern, Switzerland; yoandri11@yahoo.es (Y.H.); matthias.liniger@ivi.admin.ch (M.L.); obdulio.garcia-nicolas@ivi.admin.ch (O.G.-N.); markus.gerber@ivi.admin.ch (M.G.)
2 Department of Infectious Diseases and Pathobiology (DIP), University of Bern, 3012 Bern, Switzerland
3 Graduate School for Cellular and Biomedical Sciences (GCB), University of Bern, 3012 Bern, Switzerland
4 Centro Nacional de Sanidad Agropecuaria (CENSA), San José de las Lajas 32700, Cuba; friaslepoureau@gmail.com (M.T.F.); claura@censa.edu.cu (C.L.P.)
5 WOAH Reference Laboratory for Classical Swine Fever, IRTA-CReSA, 08193 Barcelona, Spain; s.munoz@vidium-solutions.com (S.M.-G.); liani.coronado@irta.cat (L.C.); llilianne.ganges@irta.cat (L.G.)
6 Unitat Mixta d'Investigació IRTA-UAB en Sanitat Animal, Centre de Recerca en Sanitat Animal (CReSA), 08193 Barcelona, Spain
7 IRTA, Programa de Sanitat Animal, Centre de Recerca en Sanitat Animal (CReSA), 08193 Barcelona, Spain
* Correspondence: nicolas.ruggli@ivi.admin.ch

Table S1. Oligonucleotides used to amplify six overlapping PCR fragments for sequencing and assembly of the Margarita cDNA (5′ and 3′ ends determined by RACE, see Materials and Methods).

Fragment (size)	Position[1]	Primer name	Primer sequence[2]
F1 (2590bp)	228-246	P-228-F	GTGGACGAGGGCATGCCCA
	2798-2817	gvPdR-R4	TAGAAAGCACTACCGTTCAG
F2 (2393 bp)	2266-2285	gvPdR-F3	GTTATTTGAAGAGGCAGAAC
	4639-4658	gvPdR-R7	TGACAACGAAGAAGTTAGAG
F3 (2999 bp)	3509-3528	P-3509-F	CTGTGGCTAATAGTGACCTA
	6488-6507	P-6509-R	GATTCTATCGCGTTGGTTGC
F4 (2861 bp)	6221-6240	P-6221-F	GGGCAGAAACACCCTATAGA
	9062-9081	P-9082-R	GTTATTCTTGTGTACCAATTC
F5 (3289 bp)	8750-8769	P-8750-F	CCAGTGATAAGAATGGAAGG
	12019-12038	gvPdR-R17	TGACTCTCAGCCTCCTTAAC
F6 (1796 bp)	10497-10516	gvPdR-F15	TGAAACGACCCGAGTTAGAG
	12270-12292	P-12292-R	TTAGGAAATTTACCTTAGTCCAAC

[1] Position in the Margarita genome, GenBank accession number [pending; submission ID 2778143].
[2] Sequence based on the PdR sequence, GenBank accession number KX576461. Retrospective mismatches with the PdR sequence are underlined.

Table S2. CSFV complete genome sequences selected for the phylogenetic analysis of Figure 1b

GenBank accession number [1]	CSFV strain [2]	Subgenotype [2]
X87939.1	Alfort/187	1.1
AF092448.2	Shimen	1.1
AY259122.1	Riems	1.1
M31768.1	Brescia	1.2
KJ873238.1	Rovac	1.2
AY578688.1	RUCSFPLUM	1.2
MG655308.1	Guatemala	1.3
OR997840.1	CSFV/Margarita/1958	1.4
KX576461.1	CSFV/1.4/dp/CSF1058/2010/Pinar del Rio	1.4
GQ923951.1	SXCDK	2.1
HQ148063.1	CSFV/2.1/dp/CSF1048/2009/LT/Penevezys	2.1
KY290453.1	CSFV/swine/S.Korea/YC16CS/2016	2.1
JX218094.1	HNSD-2012	2.1
MH979231.1	HY78	2.1
JQ861548.3	CSFV-UP-GZ-NVD-11	2.2
KC851953.1	CSFV_IND/UK/LAL-290	2.2
MH979232.1	ND20	2.2
GU233731.1	CSFV/2.3/dp/CSF857/2006/Borken	2.3
GU233733.1	CSFV/2.3/wb/CSF1046/2009/Hennef	2.3
GU324242.1	CSFV/2.3/wb/XXX0609/2004/Uelzen	2.3
HQ148062.1	CSFV/2.3/dp/CSF864/2007/BG/Jambul	2.3
KF669877.1	JJ9811	3.2
KT716271.1	YI9908	3.2
AY646427.1	94.4/IL/94/TWN	3.4
L49347.1 [3]	P97	3.4

[1] Version used in the phylogenetic tree

[2] The CSFV strains were selected to cover the three genotypes and the major subgenotypes for which complete genome sequences were available.

[3] Incomplete 5′ and 3′ ends

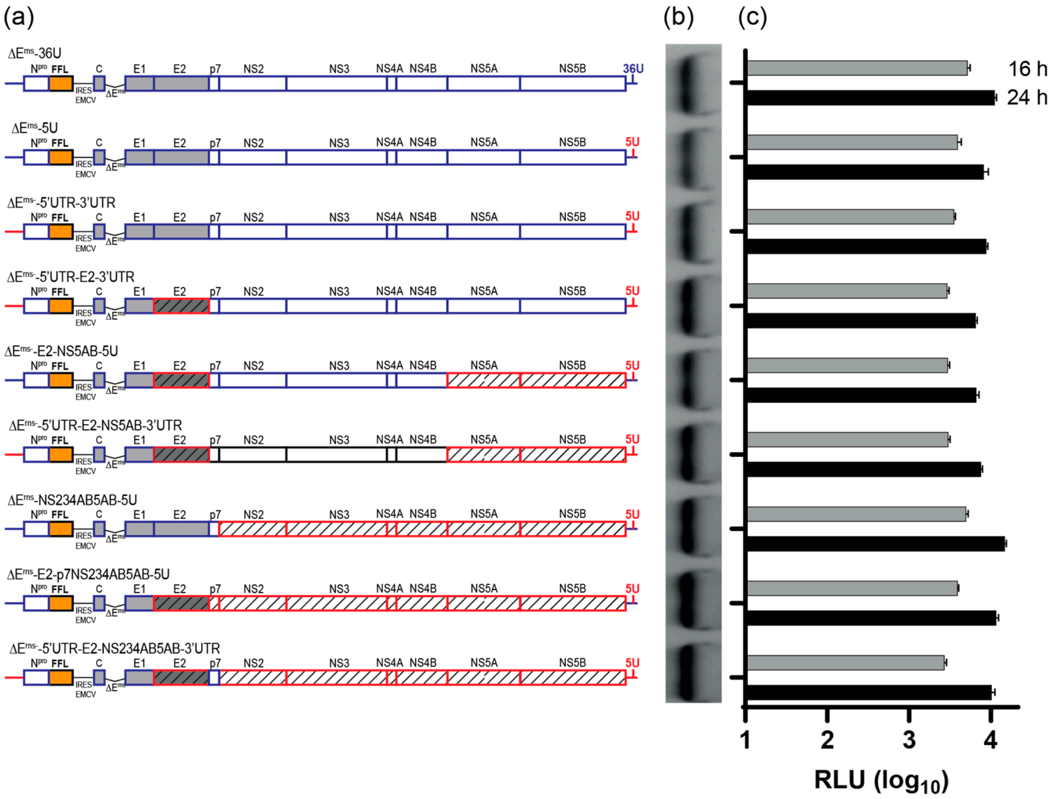

Figure S1. Firefly luciferase (FFL) expression from replicons of PdR (ΔErns-36U) and Margarita (ΔErns-5′UTR-E2-NS234AB5AB-3′UTR) and from chimera thereof in PEDSV.15 cells does not reflect the difference of virulence observed *in vivo*. **(a)** Schematic representation of the bicistronic replicons encoding FFL and lacking the Erns gene (ΔErns) in the second ORF downstream of the internal ribosomal entry site of encephalomyocarditis virus (IRES EMCV), with gene elements from vMargarita (red) in the PdR backbone (blue). **(b)** Analysis of T7 *in vitro* transcripts (0.5 µg/lane) of the replicons by agarose gel electrophoresis. **(c)** FFL activity of each replicon at 16- and 24-hours post-transfection of PEDSV.15 cells. FFL was normalized for transfection efficiency by flow cytometry against E2 protein. Bars represent the mean of triplicate transfections, with error bars showing the SD.
